# gQuant, an Automated Tool for Quantitative Glycomic Data Analysis

**DOI:** 10.3389/fchem.2021.707738

**Published:** 2021-07-28

**Authors:** Jiangming Huang, Biyun Jiang, Mingqi Liu, Pengyuan Yang, Weiqian Cao

**Affiliations:** ^1^ The Fifth People’s Hospital, Fudan University, and the Shanghai Key Laboratory of Medical Epigenetics, The International Co-laboratory of Medical Epigenetics and Metabolism, Ministry of Science and Technology, Institutes of Biomedical Sciences, Fudan University, Shanghai, China; ^2^ Department of Chemistry, Fudan University, Shanghai, China; ^3^ NHC Key Laboratory of Glycoconjugates Research, Fudan University, Shanghai, China

**Keywords:** glycomic quantitation, stable isotope labeling, MALDI-MS analysis, quantitative tool, automated processing

## Abstract

MALDI-MS-based glycan isotope labeling methods have been effectively and widely used for quantitative glycomics. However, interpretation of the data produced by MALDI-MS is inaccurate and tedious because the bioinformatic tools are inadequate. In this work, we present gQuant, an automated tool for MALDI-MS-based glycan isotope labeling data processing. gQuant was designed with a set of dedicated algorithms to improve the efficiency, accuracy and convenience of quantitation data processing. When tested on the reference data set, gQuant showed a fast processing speed, as it was able to search the glycan data of model glycoproteins in a few minutes and reported more results than the manual analysis did. The reported quantitation ratios matched well with the experimental glycan mixture ratios ranging from 1:10 to 10:1. In addition, gQuant is fully open-source and is coded in Python, which is supported by most operating systems, and it has a user-friendly interface. gQuant can be easily adapted by users for specific experimental designs, such as specific glycan databases, different derivatization types and relative quantitation designs and can thus facilitate fast glycomic quantitation for clinical sample analysis using MALDI-MS-based stable isotope labeling.

## Introduction

Protein glycosylation plays significant roles in many biological and physiological processes, including cell adhesion, sperm fusion, and protein folding, as well as in protein half-life (Hart and Copeland, 2010; Xu and Ng, 2015). It has also been reported that aberrant glycosylation has a substantial impact on host-cell reactions, infectious diseases such as tuberculosis, the progression of tumors (Lu et al., 2016), autoimmune diseases (Eakin et al., 2016) and prostate cancer (Shah et al., 2015). Therefore, precision quantitation of glycans is greatly needed to better understand glycan functionality, measure different glycan levels and potentially discover glycan biomarkers.

Many efforts have been made to develop mass spectrometry (MS)-based glycan quantitation techniques due to the excellent qualitative ability, sensitivity and high throughput of mass spectrometry (Wuhrer, 2013; Cao et al., 2020). For example, a metabolic labeling strategy was introduced for *in vivo* labeling and glycan quantitation (Orlando et al., 2009); chemical labeling strategies using different isotope labeling reagents, such as 2-aminobenzoic acid, aniline, arginine, and 1-phenyl-3-methyl-5-pyrazolone, were developed for glycan derivatization and quantitation (Prien et al., 2010; Albrecht et al., 2017; Cai et al., 2015; Smith et al., 2017; Wang et al., 2017); and enzymatic ^18^O labeling was also utilized for glycan relative quantification (Zhang et al., 2015; Cao et al., 2015). Among the developed techniques, MALDI-MS-based methods have shown high feasibility, efficiency and speed in quantitative glycan analysis and have been widely used. However, MALDI-MS-based glycan quantitation data have mostly been processed manually. This work is tedious and requires expert knowledge of protein glycosylation. Thus, this drawback greatly impedes the development of quantitative glycomics and the understanding of protein glycosylation.

Some studies have been carried out to develop bioinformatic tools to assist glycan MS data interpretation and quantitation. GlycoWorkbench is one of the most popular glycomic analytical tools that can search glycans with given m/z(s). However, it lacks quantitation modules (Ceroni et al., 2008). GlycoReSoft was developed to help annotate and quantitate glycans, but it can only be applied for LC-ESI-MS data (Maxwell et al., 2012). Massytools was mainly designed to quantitate the abundance of glycans from the total sample or monoclonal antibody and report normalized percentage results (Jansen et al., 2015), while LaCytools was designed for LC-ESI-MS-based targeted glycan quantitation (Jansen et al., 2016). Multiglycan was introduced to obtain glycan mass spectra from MALDI-MS or LC-ESI-MS. Multiglycan detects different combination modes of glycans and can be used for glycan quantitation (Yu et al., 2013a; Hu et al., 2015). Although these tools were designed for glycan searches and to assist quantitation (Yu et al., 2013b), the glycan quantitation functions remain underdeveloped, especially for stable isotope labeling-based glycan relative quantitation data. Therefore, a precision and user-friendly tool for glycan isotope labeling-based MS data analysis is still needed.

In this work, we developed a MALDI-MS-based relative quantitation glycan data processing tool, gQuant. gQuant is embedded with well-defined glycan databases and a set of thoroughly designed algorithms to facilitate automated MALDI-MS-based glycan data preprocessing, glycan identification and quantitation ratio calculations. Glycan isotope patterns were studied to aid the precise quantitation of biological samples. Moreover, gQuant was developed using the Python 2.7 and Python 3 programming language and can be used in most operating systems. gQuant is a fully open-source software tool that is fast and has a user-friendly interface to assist glycan MS data processing.

## Materials and Methods

The development of the gQuant tool for automated glycan quantitation data processing includes two sections: the preparation of a comprehensive glycan database and establishment of the quantitation tool. A set of MS data was further analyzed to test the feasibility of the newly developed glycan tool. Detailed construction and testing of gQuant are listed below.

### Glycan Database Preparation

The inherent glycan database in gQuant was constructed by carefully collecting and integrating information from published data and literatures (Ranzinger et al., 2009; Balog et al., 2012; and; Liu et al., 2014). As reported, glycans from human sources rarely contain N-glycolylneuraminic acid (NeuGc). Therefore, two glycan databases, human-sourced and mammalian-sourced, were developed and contained 344 and 419 nonredundant glycans, respectively. The largest difference between the two databases is that the mammalian glycan database is composed of both N-acetylneuraminic acid (NeuAc) and N-glycolylneuraminic acid (NeuGc), while sialyated glycans in the human glycan database is composed of only N-acetylneuraminic acid (NeuAc). Element compositions, glycans and corresponding molecular weights (both residual and free-ended) were uniform, calculated and well recorded in the database file. In addition, distinct glycan isotope distributions were generated and recorded *via* the online tool MS-ISOTOPE (https://prospector2.ucsf.edu/prospector/cgi-bin/msform.cgi?form=msisotope). The isotope distributions were further applied for isotope interference calculations to increase quantitation accuracy. It should be noted that such a glycan database can be well extended or customized to perform, for example, the incorporation of O-glycans according to users’ needs.

### gQuant Tool Development

gQuant was created with a series of algorithms, including spectral preprocessing, glycan mapping, quantitation and ratio calculations. For profiled data, peak picking and centroiding algorithms were applied using Gaussian distribution fitting adapted from pymzML (Kösters et al., 2018). The noise level was also calculated by means of the “median” or “mean” value of the spectra multiplied by a predefined coefficient to facilitate signal-to-noise level estimation and signal filtration. Spectra were then deisotoped, and peaks without sufficient isotopes (by default, the envelope should contain more than three isotopes) were discarded. All satisfied isotope envelopes were recorded and transferred for further glycan matching and quantitation.

The glycan database was also customized for all given charge carriers (adducts, for example; H+, Na+, K+, et al. in positive mode and -H, Na-2H, K-2H, et al. in negative mode) and glycan derivatives (for example, 2-AB, reducing end, or nonderivatized, which was named “free end” in gQuant). After spectral preprocessing and database customization, a molecular-weight-based glycan exhaustive match algorithm was applied to all monoisotopic peaks in the spectra. All possible glycan molecular forms (adducts, derivatizations) were recorded. For stable isotopic labeling-based glycan quantitation data, heavily labeled glycans were also searched, and each matched glycan (light isotopic labeled and heavy isotopic labeled) was tagged with “L” and “H”, respectively, in the final results.

If the parameter of delta mass was not defined (set as zero by default), gQuant only output matched glycans, peaks and corresponding peak intensities, and the results can assist users in determining all detected glycans or calculating normalized percentages. If delta mass was not zero, the final glycan relative quantitation ratio calculation was calculated in the tool by Eq. 1 and Eq. 2 as shown below:

$${\text{Ratio}}_{\text{calibr}}=\frac{{\text{Intensity}}_{\text{light}}}{{\text{Intensity}}_{\text{heavy}}-{\text{Intensity}}_{\text{light}}\times {\text{calibr}}_{\text{ratio}}}$$ (1)

$${\text{calibr}}_{\text{ratio}}=\frac{\text{Theoretical}\;{\text{Abundance}}_{\text{isotope}\;\text{for}\;\text{quantitation}+\text{delta}\;\text{mass}}}{\text{Theoretical}\;{\text{Abundacne}}_{\text{isotope}\;\text{for}\;\text{quantitation}}}$$ (2)

where delta mass was the round number of delta mass.

To facilitate the usage of gQuant, a user-friendly interface (UI) was also implemented. Detailed information on the UI is presented in the *Results and Discussion* section and Supplementary material 1.

### Feasibility Test of gQuant

A series of datasets (GREDIL and PFBHA datasets) were applied to evaluate the performance of gQuant. The GREDIL dataset used NaBH_4_ to reduce PNGase F-released glycans and incorporated ^18^O + deuterium to form a delta mass of 3.0 Da between samples, while the PFBHA dataset used o-(2,3,4,5,6-pentafluorobenzyl) hydroxylamine hydrochloride (PFBHA) and PFBHA-2 deuterium for sample glycan derivatization. Detailed methods of the two datasets were well described in previous publications (Cao et al., 2015; Yang et al., 2019). The datasets were adopted as part of the following search parameters. For the PFBHA dataset, raw data were preprocessed by the vendor-provided software Data Explorer 4.3 (AB SIEX, Framingham, MA, United States). Briefly, centroid peaks were generated with the *Centroiding* function in the *Process* menu. Peak lists with m/z and intensity were then exported to standalone ASCII formatted data files via *File* menu→*Export*→*ASCII Spectrum*→*Save*. For the GREDIL dataset, since the vendor software failed to centroid peaks properly, profiled form data were exported by the Shimadzu Biotech Launchpad MALDI-MS application 2.9.3 (Shimadzu, Japan) *via File* menu→*Export*→*ASCII … →* with *Processed* selected and saved. All data files were then analyzed with the gQuant tool with the m/z tolerance set to 50ppm, adducts of H+, Na+, K+ considered, a positive mode, max charge of 3, signal-to-noise ratio set as 10 and derivatization type and delta masses set according to corresponding experimental designs.

## Results and Discussion

**FIGURE 1:**
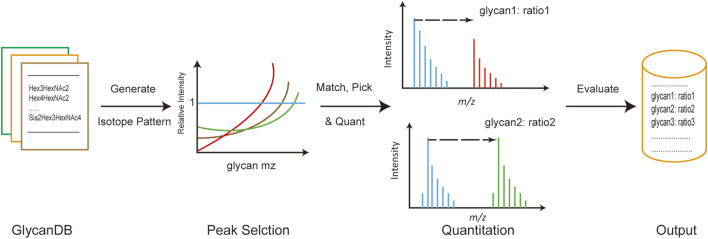
General pipeline of gQuant for MS-based glycan quantitation using stable isotope labeling.

The general process of developing the gQuant tool is depicted in Figure 1. Briefly, integrated glycan databases for human and mammalian samples were carefully collected and extracted from the current glycan database and published literature. As a result, a total of 419 glycan compositions were recorded in the mammalian glycan database, with maximum hexose (Hex), N-acetylhexosamine (HexNAc), N-acetylneuraminic acid/N-glycolylneuraminic acid (NeuAc/NeuGc), and fucose (dHex) values of 12, 7, 4, and 5, respectively, and 344 entries were recorded in the human glycan database (No NeuGc). The maximum glycan molecular weight of the database was approximately 4,500 Da, which covered the most common glycans (Supplementary Table 1 and Supplementary Table 2). Chemical element compositions of each glycan were then generated, and the theoretical isotope envelopes for each composition were calculated. Peak lists were obtained by matching the isotope envelopes with the theoretical glycan m/z under predefined mass tolerance, derivatizations, charge carriers and isotopic labeling mass (according to experimental designs and instrument types). Then, annotated peaks were quantitated by gQuant, and the quantitation ratio (heavy to light) was reported.

**FIGURE 2:**
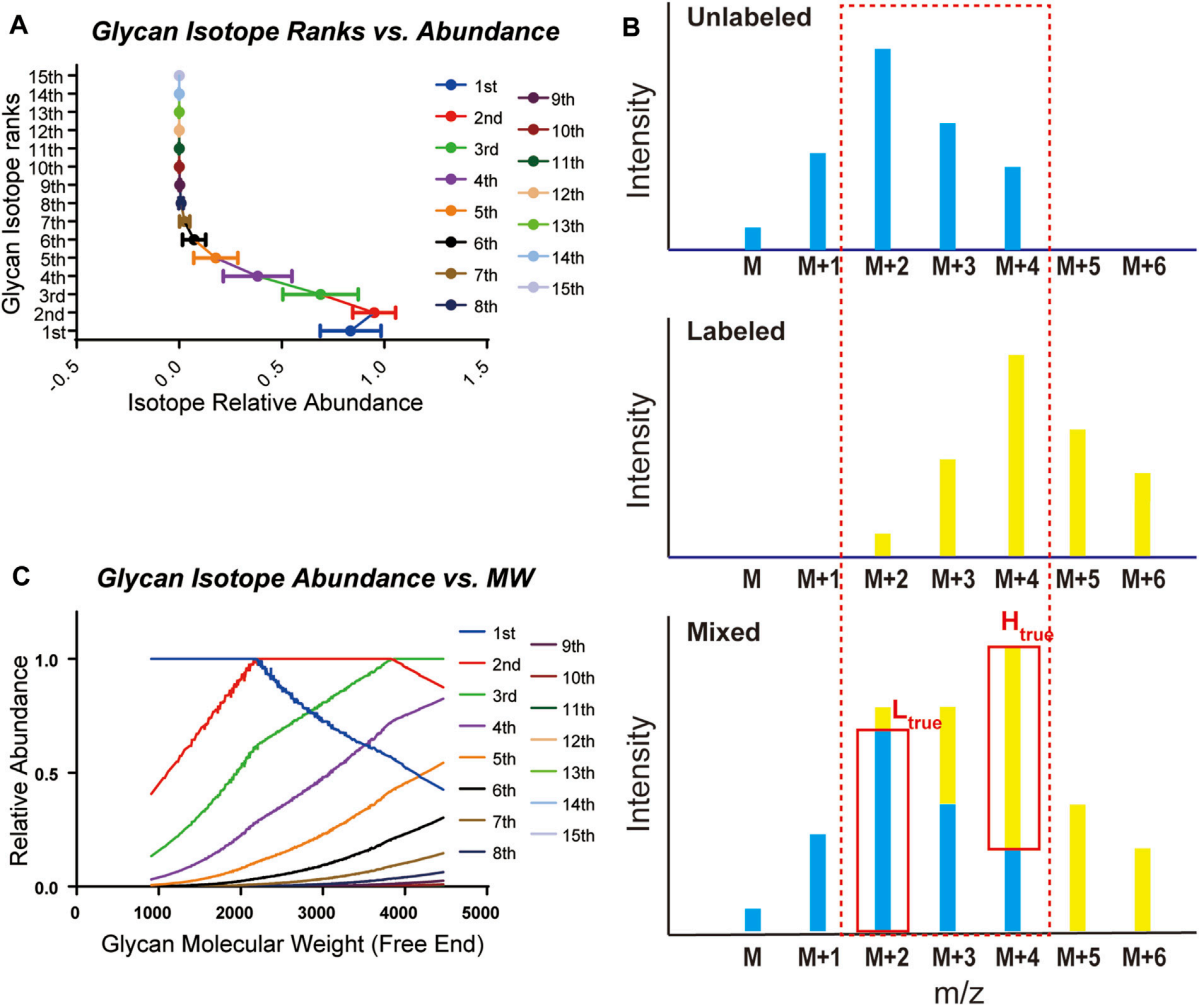
Statistical analysis of glycan isotopic patterns used in gQuant. **(A)** Theoretical relative abundance of all 15 isotopes in the mammalian glycan database (maximum isotope abundance in each glycan was set as 1.0). **(B)** Ratio calibration considering isotope co-interference in quantitation. **(C)** Distribution of theoretical relative intensity of glycan isotopes.

Glycans are composed mainly of C, H, N, and O elements and show distinct isotopic patterns. Isotopic patterns can be further utilized to assist glycan identification and quantitation. Therefore, it is important to study the isotopic patterns of glycans to better understand glycan mass spectrometry behaviors such as isotope envelopes. Statistical investigation of glycan isotopic patterns benefited from the well-constructed mammalian glycan database. As shown in Supplementary Table 1 and Supplementary Table 2, by theoretical calculation of glycan elemental compositions, glycans in the gQuant database reported a maximum of 14 isotopes with abundance. In addition, it can be concluded that the eighth isotopes accounts for less than 1% of the highest isotopes (Figure 2A, Supplementary Table 3); therefore, a mass interval of higher than 8 Da is normally sufficient to ensure avoidance of glycan isotope interference. For mass intervals smaller than 8 Da, the isotope pattern in the gQuant can be applied to recalibrate the quantitation ratio, as shown in Figure 2B and Equation (2) described in Methods part. As glycan molecular weight increased, the highest isotope ion peak gradually changed from the first isotope ion peak to the second one at approximately 2,250 and to the third one at 3,850 (Figure 2C). Since glycans are highly polar and fragment poorly in MS, it would be meaningful to use more abundant isotopes for quantitation to increase quantitation sensitivity and accuracy. The features have already been incorporated in gQuant to improve the performance of glycan quantitation.

The accuracy and feasibility of gQuant was assessed by quantitating PFBHA datasets (Yang et al., 2019), in which glycans from model glycoproteins were derivatized with PFBHA or PFBHA-2 deuterium. As shown in Supplementary Table 4, glycan derivatives were almost always found in the top-ranked results, as the matched mass tolerances were in ascending order. In addition, the reported ratios in the L/H ratio column were close to 1.0, as experimentally designed. In addition to quantitation ratios, gQuant can also report other information, such as glycan compositions, glycan derivatization types, charge carriers, paired m/z values and intensities (Supplementary Table 5), thus, greatly facilitating the further collection and understanding of quantitative glycan information. Based on the detailed analysis of the theoretical glycan isotope distribution, the highest isotope-based selection for the quantitation strategy was applied in gQuant to enhance glycan quantification. It should be noted that only the molecular weight is used for glycan matching; therefore, gQuant reports all matched glycans. For further filtration of suspicious identifications, we suggest setting proper mass tolerance according to different mass spectrometry instruments and strategies they used. For example, the MS tolerance of 50ppm is recommended for the PFBHA datasets. Further manual check or even tandem mass spectrometry verification may be necessary for some target candidates or some ambiguous results as appropriate.

**FIGURE 3:**
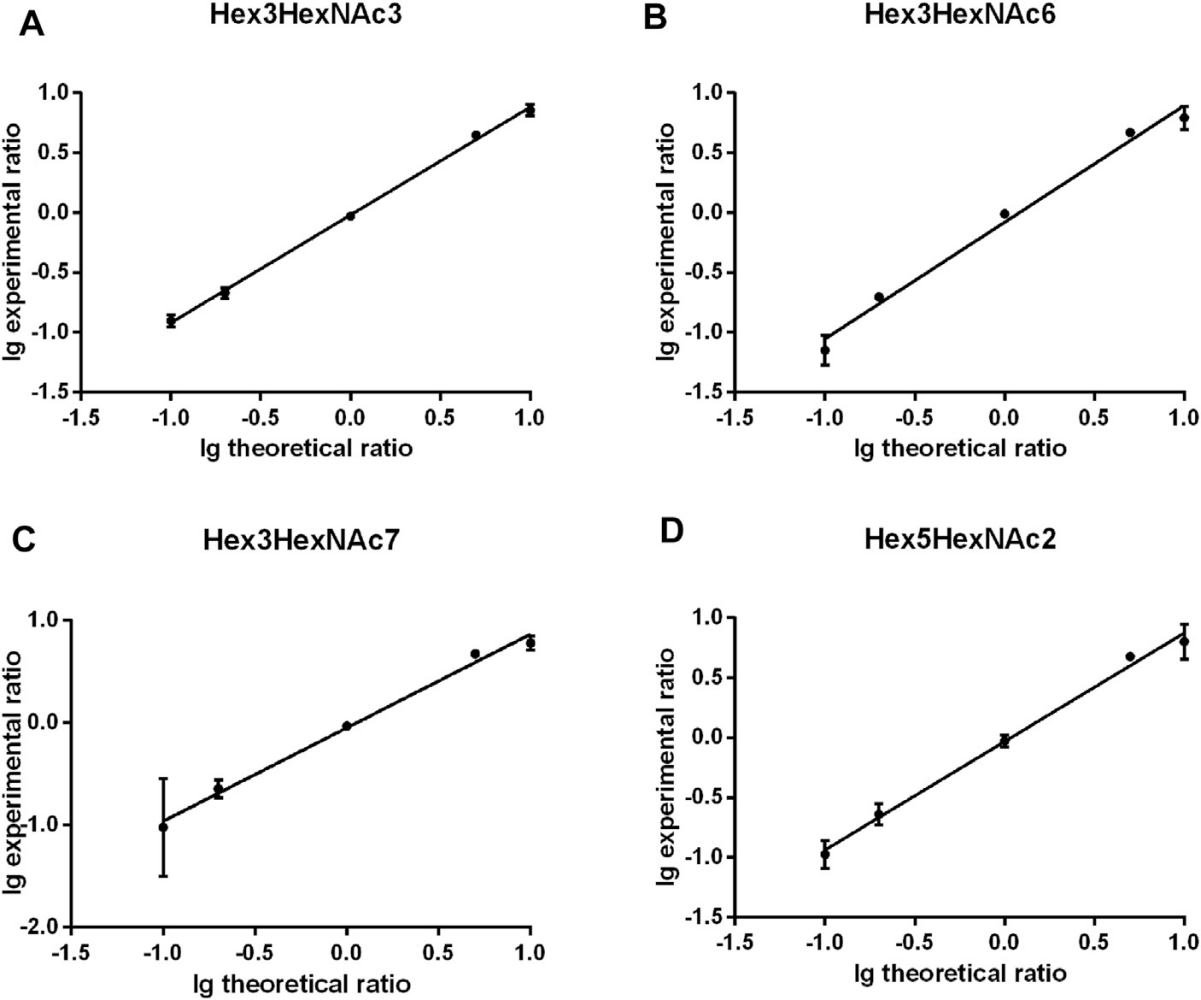
Comparison of gQuant the reported ratio with experimental mixed ratios (ranging from 10:1, 5:1, 1:1 1:5, and 1:10) of four glycans. **(A)** Hex3HexNAc3, **(B)** Hex3HexNAc6, **(C)** Hex3HexNAc7 and **(D)** Hex5HexNAc2.

The quantitation performance of gQuant was further evaluated on differentially labeled glycans with defined ratios (heavy labeled and unlabeled samples with a mass shift of 2.0 Da (2 deuterium vs 2 hydrogen) mixed at 10:1, 5:1, 1:1, 1:5, and 1:10, v/v, respectively) from PFBHA datasets. As shown in Figure 3 and Supplementary Table 6, the quantitation ratios reported by gQuant were consistent with the experimental designs and manual analytical results for four glycans (Hex3HexNAc3 [M + Na]^+^ 1,331.40601, Hex3HexNAc6 [M + Na]^+^ 1940.64413, Hex3HexNAc7 [M + Na]^+^ 2,143.7235, and Hex5HexNAc2 [M + Na]^+^ 1,452.43229). In addition, gQuant reported more glycans (37 glycans, Supplementary Table 4) than manually did (only two glycans were reported). It was also observed that there could be a fixed mass offset in different tests due to the instrumental errors. To reduce the mass shift error, gQuant also offered a simple parameter of calibrated mass to recalibrate the mass error and improve the matching and quantitating accuracy. In addition to test on quantifying glycans from single glycoprotein, gQuant was further applied to quantitation of glycans from a complex sample, rat serum, the data of which were obtained from GREDIL dataset (Cao et al., 2015). As shown in Supplementary Table 7, a total of 15 glycan peaks with calibrated mass, such as m/z 935.39 Hex3HexNAc2 Na^+,^ m/z 1,665.44 Hex5HexNAc4 Na+/Hex4HexNAcdHex1 K^+^, were identified and quantified. The reported glycan quantitation ratio was closed to mixed ratio. Identified glycans were relatively consistent with previous publication (Cao et al., 2015). Thus, gQuant showed high accuracy and was informative for glycan quantitation data processing.

The efficiency of gQuant was also tested on the aforementioned dataset. As shown in Supplementary Table 8, it took less than 3 min to process a single model protein data file and less than 25 min for complex samples, while it took hours or days to process sample data manually. In addition, gQuant supports the batch running mode, and the running speed can be further accelerated by algorithm optimization for the glycan matching process in the future. Thus, because it is automated and efficient, gQuant can greatly improve the speed and throughput of glycan quantitation analysis.

**FIGURE 4:**
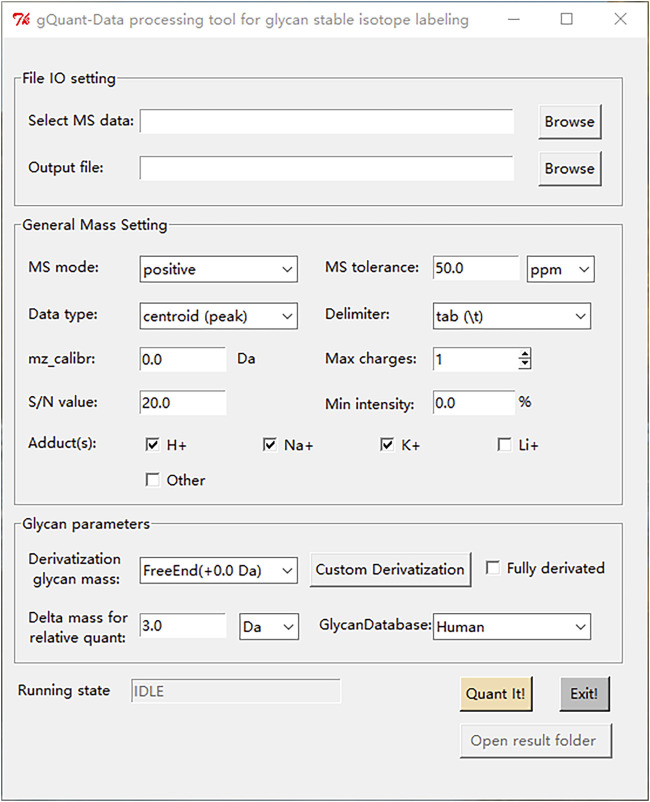
The graphical user interface of gQuant.

The common obstacle for the application of most bioinformatic tools is the lack of user-friendly interfaces. To facilitate the usage of gQuant, a convenient user interface was designed and implemented, as shown in Figure 4. There are three main panels of gQuant UI: a data file input and output (IO) setting panel, a mass spectrometry parameter configuration panel (*General Mass Setting*), and a glycan related parameter setting panel (*Glycan Parameters*). The data file input and output setting panel enables batched MS data selection and output directory settings; the *General Mass Setting* panel contains parameters such as MS mode (positive or negative), data point types (profile, or centroid), signal-to-noise level setting, charge carrier setting (adducts) checkboxes, etc.; the *Glycan Parameters* panel facilitates glycan derivatization setting either by predefined items (2-AB, reducing end, etc.,) or to define a new derivatization type by “Custom Derivatization”; and it also supports delta mass setting for relative quantitation and glycan database selection. In addition, as mentioned above, gQuant is programmed with Python 2.7 and Python 3, which is expected to be supported by most operating systems, such as Windows, Mac or Linux. Moreover, gQuant is fully open-source software under the Apache 2.0 license, and it can be easily adapted to accommodate different analytical purposes, for example, to modify the glycan database for different sourced samples or for O-glycan analysis, or to add a function to support LC-MS quantitation data analysis.

## Conclusion

Glycan quantitation MS data processing remains a tedious and challenging task for researchers. To tackle this problem, we designed and implemented an automated glycan quantitation tool, gQuant, in this work. gQuant is capable of automatically and efficiently processing quantitative glycan mass spectrometry data and reporting all matched glycans and quantitation ratios. gQuant was embedded in N-glycan databases for human (No NeuGc)- or mammalian (Containing NeuGc)-sourced samples. Statistical evaluation of glycan isotopic distributions was performed to study the distinct isotopic patterns of glycans. It was suggested that the glycan isotope interference decreased to less than 1% when the isobaric interval increased above 8 Da gQuant enables automated glycan matching, glycan abundance ratio calculation and data output. Compared with manual interpretation, this tool showed good accuracy and efficiency. The performance test indicated that gQuant can successfully process glycan quantitation data and report precision ratios, that it reports more glycans than the manual method and that it is much more efficient than the manual method (with a speed as low as several minutes). Although the presented results are all from N-glycan data, gQuant can also be easily adapted for relative quantitation of other types of glycans, such as mucin-type O-glycans, with a specified or user-defined glycan database. In summary, as an open-source, convenient and efficient software tool, gQuant is expected to facilitate glycomic studies and clinical glycan MS data analysis.

## Data Availability

The original contributions presented in the study are included in the article/Supplementary Material, further inquiries can be directed to the corresponding author.

## References

[B1] Albrecht S. Mittermayr S. Smith J. Martín S. M. Doherty M. Bones J. (2017). Twoplex 12/13 C6 Aniline Stable Isotope and Linkage-Specific Sialic Acid Labeling 2D-LC-MS Workflow for Quantitative N-Glycomics. Proteomics 17 (1-2), 1600304. 10.1002/pmic.201600304 27891772

[B2] Balog C. I. A. Stavenhagen K. Fung W. L. J. Koeleman C. A. McDonnell L. A. Verhoeven A. (2012). N-glycosylation of Colorectal Cancer Tissues. Mol. Cell Proteomics 11 (9), 571–585. 10.1074/mcp.m111.011601 22573871 PMC3434767

[B3] Cai Y. Jiao J. Bin Z. Zhang Y. Yang P. Lu H. (2015). Glycan Reductive Isotope-Coded Amino Acid Labeling (GRIAL) for Mass Spectrometry-Based Quantitative N-Glycomics. Chem. Commun. 51 (4), 772–775. 10.1039/c4cc08086f 25421075

[B4] Cao W.-Q. Liu M.-Q. Kong S.-Y. Wu M.-X. Huang Z.-Z. Yang P.-Y. (2020). Novel Methods in Glycomics: a 2019 Update. Expert Rev. Proteomics 17 (1), 11–25. 10.1080/14789450.2020.1708199 31914820

[B5] Cao W. Zhang W. Huang J. Jiang B. Zhang L. Yang P. (2015). Glycan Reducing End Dual Isotopic Labeling (GREDIL) for Mass Spectrometry-Based Quantitative N-Glycomics. Chem. Commun. 51 (71), 13603–13606. 10.1039/c5cc05365j 26240031

[B6] Ceroni A. Maass K. Geyer H. Geyer R. Dell A. Haslam S. M. (2008). GlycoWorkbench: a Tool for the Computer-Assisted Annotation of Mass Spectra of Glycans. J. Proteome Res. 7 (4), 1650–1659. 10.1021/pr7008252 18311910

[B7] Eakin A. J. Bustard M. J. McGeough C. M. Ahmed T. Bjourson A. J. Gibson D. S. (2016). Siglec-1 and -2 as Potential Biomarkers in Autoimmune Disease. Prot. Clin. Appl. 10 (6), 635–644. 10.1002/prca.201500069 26752092

[B8] Hart G. W. Copeland R. J. (2010). Glycomics Hits the Big Time. Cell 143 (5), 672–676. 10.1016/j.cell.2010.11.008 21111227 PMC3008369

[B9] Hu Y. Zhou S. Yu C.-Y. Tang H. Mechref Y. (2015). Automated Annotation and Quantitation of Glycans by Liquid Chromatography/electrospray Ionization Mass Spectrometric Analysis Using the MultiGlycan-ESI Computational Tool. Rapid Commun. Mass. Spectrom. 29 (1), 135–142. 10.1002/rcm.7093 25462374 PMC4516131

[B10] Jansen B. C. Falck D. de Haan N. Hipgrave Ederveen A. L. Razdorov G. Lauc G. (2016). LaCyTools: A Targeted Liquid Chromatography-Mass Spectrometry Data Processing Package for Relative Quantitation of Glycopeptides. J. Proteome Res. 15 (7), 2198–2210. 10.1021/acs.jproteome.6b00171 27267458

[B11] Jansen B. C. Reiding K. R. Bondt A. Hipgrave Ederveen A. L. Palmblad M. Falck D. (2015). MassyTools: A High-Throughput Targeted Data Processing Tool for Relative Quantitation and Quality Control Developed for Glycomic and Glycoproteomic MALDI-MS. J. Proteome Res. 14 (12), 5088–5098. 10.1021/acs.jproteome.5b00658 26565759

[B12] Kösters M. Leufken J. Schulze S. Sugimoto K. Klein J. Zahedi R. P. (2018). pymzML v2.0: Introducing a Highly Compressed and Seekable Gzip Format. Bioinformatics (Oxford, England) 34 (14), 2513–2514. 10.1093/bioinformatics/bty046 29394323

[B13] Liu M. Zhang Y. Chen Y. Yan G. Shen C. Cao J. (2014). Efficient and Accurate Glycopeptide Identification Pipeline for High-Throughput Site-Specific N-Glycosylation Analysis. J. Proteome Res. 13 (6), 3121–3129. 10.1021/pr500238v 24766575

[B14] Lu L. L. Chung A. W. Rosebrock T. R. Ghebremichael M. Yu W. H. Grace P. S. (2016). A Functional Role for Antibodies in Tuberculosis. Cell 167 (2), 433–443. 10.1016/j.cell.2016.08.072 27667685 PMC5526202

[B15] Maxwell E. Tan Y. Tan Y. Hu H. Benson G. Aizikov K. (2012). GlycReSoft: a Software Package for Automated Recognition of Glycans from LC/MS Data. PloS one 7 (9), e45474. 10.1371/journal.pone.0045474 23049804 PMC3458864

[B16] Orlando R. Lim J.-M. Atwood J. A. 3rd Angel P. M. Fang M. Aoki K. (2009). IDAWG: Metabolic Incorporation of Stable Isotope Labels for Quantitative Glycomics of Cultured Cells. J. Proteome Res. 8 (8), 3816–3823. 10.1021/pr8010028 19449840 PMC4141490

[B17] Prien J. M. Prater B. D. Qin Q. Cockrill S. L. (2010). Mass Spectrometric-Based Stable Isotopic 2-aminobenzoic Acid Glycan Mapping for Rapid Glycan Screening of Biotherapeutics. Anal. Chem. 82 (4), 1498–1508. 10.1021/ac902617t 20108906

[B18] Ranzinger R. Frank M. von der Lieth C.-W. Herget S. (2009). Glycome-DB.org: a portal for Querying across the Digital World of Carbohydrate Sequences. Glycobiology 19 (12), 1563–1567. 10.1093/glycob/cwp137 19759275

[B19] Shah P. Wang X. Yang W. Toghi Eshghi S. Sun S. Hoti N. (2015). Integrated Proteomic and Glycoproteomic Analyses of Prostate Cancer Cells Reveal Glycoprotein Alteration in Protein Abundance and Glycosylation*. Mol. Cell Proteomics 14 (10), 2753–2763. 10.1074/mcp.m115.047928 26256267 PMC4597149

[B20] Smith J. Mittermayr S. Váradi C. Bones J. (2017). & Bones, J.Quantitative Glycomics Using Liquid Phase Separations Coupled to Mass Spectrometry. Analyst 142 (5), 700–720. 10.1039/c6an02715f 28170017

[B21] Wang C. Zhang P. Jin W. Li L. Qiang S. Zhang Y. (2017). Quantitative O -glycomics Based on Improvement of the One-Pot Method for Nonreductive O -glycan Release and Simultaneous Stable Isotope Labeling with 1-(d 0/d 5 )phenyl-3-Methyl-5-Pyrazolone Followed by Mass Spectrometric Analysis. J. Proteomics 150, 18–30. 10.1016/j.jprot.2016.08.012 27585995

[B22] Wuhrer M. (2013). Glycomics Using Mass Spectrometry. Glycoconj J. 30 (1), 11–22. 10.1007/s10719-012-9376-3 22532006 PMC3547245

[B23] Xu C. Ng D. T. W. (2015). Glycosylation-directed Quality Control of Protein Folding. Nat. Rev. Mol. Cel Biol. 16 (12), 742–752. 10.1038/nrm4073 26465718

[B24] Yang L. Du X. Peng Y. Cai Y. Wei L. Zhang Y. (2019). Integrated Pipeline of Isotopic Labeling and Selective Enriching for Quantitative Analysis of N-Glycome by Mass Spectrometry. Anal. Chem. 91 (2), 1486–1493. 10.1021/acs.analchem.8b04525 30557003

[B25] Yu C.-Y. Mayampurath A. Hu Y. Zhou S. Mechref Y. Tang H. (2013b). Automated Annotation and Quantification of Glycans Using Liquid Chromatography-Mass Spectrometry. Bioinformatics (Oxford, England) 29 (13), 1706–1707. 10.1093/bioinformatics/btt190 PMC4542666 23610369

[B26] Yu C.-Y. Mayampurath A. Tang H. (2013a). Software Tools for Glycan Profiling. Methods Mol. Biol. (Clifton, N.J.) 951, 269–276. 10.1007/978-1-62703-146-2_18 PMC3861397 23296537

[B27] Zhang W. Cao W. Huang J. Wang H. Wang J. Xie C. (2015). PNGase F-Mediated Incorporation of 18O into Glycans for Relative Glycan Quantitation. Analyst 140 (4), 1082–1089. 10.1039/c4an02073a 25521995

