# Supplementary material for: gQuant, an Automated Tool for Quantitative Glycomic Data Analysis
Source: Front Chem. 2021 Jul 28;9:707738. doi: 10.3389/fchem.2021.707738 (PMC8355585; doi:10.3389/fchem.2021.707738)
Supplement: Supplementary file 4 [file DataSheet1.docx]

**Supplementary material 1. gQuant User Guide**

# **About gQuant**

- 1. gQuant was designed for automated processing of glycan quantitation data produced by MALDI-MS.

# **How does it work**

- 1. gQuant performed spectra pre-processing, glycan mapping by exhaustive algorithms and quantitation ratio calculation for matched glycans in a sequence.

# **Installation and Requirement**

- 1. gQuant was written in python 2.7 on a pythonxy spyder platform and was expected to work on most PC environment like windows (win7/win10 x64 tested), mac OS or Linux. Normally, PCs with 4 GB memory or above should meet the hardware requirements. A python 3 version gQuant was also included to facilitate users.
  2. For python 2 version, you must have Python 2.7, scipy, numpy (1.9.2), pandas (0.16.2) and tkinter installed. You can do any one of the following:

1. Download pythonxy2.7.10.0 (http://python-xy.github.io/downloads.html) (HIGHLY RECOMMENDED)
2. Download Anaconda (http://continuum.io/downloads)
3. Download Canopy Express (https://store.enthought.com/downloads/).
4. Download Python 2.7 (https://www.python.org/downloads/), and manually install the libraries
5. Python libraries can be installed via the pip package manager (HIGHLY RECOMMENDED) or easy install
6. Use **pip uninstall SomePackage** to uninstall incompatible module and reinstall distinct version module via **pip install SomePackage==1.0.4**
7. Download the gQuant *package* in a zip. Unpack the “py2” folder and save to a convenient directory.
   1. For python3 version, you must have scipy, numpy, pandas and tkinter installed. You can do any one of the following:
8. Download Anaconda (http://continuum.io/downloads)
9. Download Canopy Express (https://store.enthought.com/downloads/).
10. Download latest Python (for example 3.9.5 at https://www.python.org/downloads/), and manually install the libraries
11. Python libraries can be installed via the pip package manager (HIGHLY RECOMMENDED) or easy install
12. Use **pip uninstall SomePackage** to uninstall incompatible module and reinstall distinct version module via **pip install SomePackage==1.0.4**
13. Download the gQuant package in a zip. Unpack the “py3” folder and save to a convenient directory.

# **Parameter Setting Instructions & Usage**

- 1. Parameter Setting Instructions
     1. The *File IO setting* panel

1. The input file(s) setting can be done in the first line either by typing in the target file directory or click the “browse” button (recommend). If no files choose, an indicator (“Select correct data file!”) will be shown in the text field once click the yellow “*Quant it”* button；
2. Output setting can be can be done in the second line either by typing in the target file directory or click the “browse” button (recommend). If left, empty, output files will be named automatically according to corresponding ms data file name.


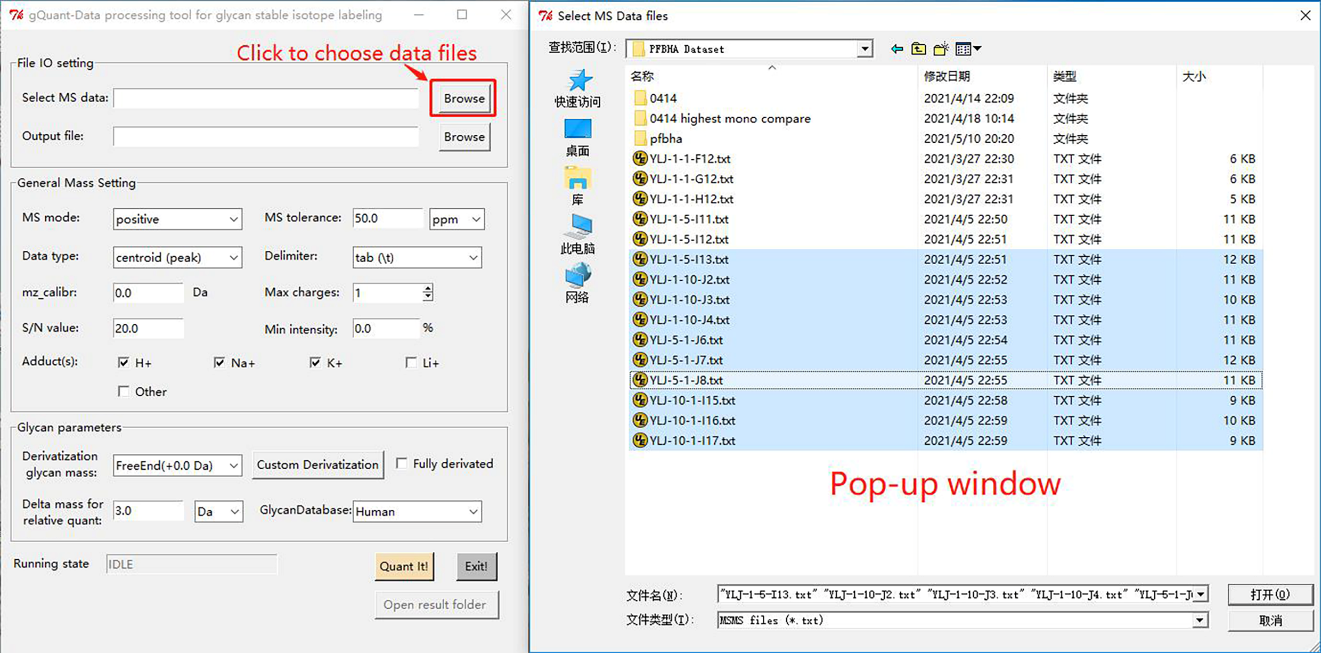


Figure S1 gQuant user interface and input file selection

- - 1. The *General Mass Setting* panel

1. Mass mode: a combo-box with positive or negative value
2. MS tolerance: a parameter used in the glycan matching and quantitation process. It contains a text filed that accepts numerical values and a unit setting that supports “ppm” and “Da”, set proper ms tolerance values according to instrument resolution.
3. Data type: set properly according to exported MS datatypes. It accepts optional “*profile(curve)”* and “*centroid(peak)”* in the combobox.
4. Delimiter: set properly according to raw MS data file export option. It supports ‘tab’, ‘comma’, ‘space’ and ‘semicolon’ in the combobox, for exported data with other delimiter, kindly contact us to include.
5. mz_calibr: a parameter to recalibrate mass shifts in the spectra pre-processing step (see equation below); it accepts positive and negative numerical values and a fixed unit of Dalton. By default, mz_calibr was set 0.0 Da.

$${m/z}_{calibrated}= {m/z}_{original}+mz\_calibr$$

6. Max charge: highest charge levels to be considered, a spinbox with value from 1 to 8 is provided. Normally for MALDI-MS, 1 is recommended.
7. S/N value: signal to noise ratio, *only used for profiled data type in gQuant*. The noise level was calculated by averaging the total intensity.
8. Min intensity: used to filter out low intensity peaks as specify
9. Adducts: charge carrier setting as desired
   - 1. The *General Mass Setting* panel
10. Derivatization glycan mass: provide some default derivatization such as reduced end, 2-AB, et al. Users can also build new derivatization by click on the button “Custom Derivatization” and a new text field and value input will be shown.
11. Full derivatized: if checked, only fully derivatized glycan form considered.
12. Delta mass for relative quant: parameter to input isobaric interval for relative quantitation.
13. GlycanDatabase: provides Human and Mamalian glycan database as instructed in the article.

4.2 For windows usage

1. Open the tool folder containing gQuant
2. Double click Run_gQuant.bat
3. Please wait patiently before user interface prompt
4. Fill in necessary information as instructed in the UI and click yellow button at the left bottom to start data processing.
5. Please wait patiently before data processing finished. In our test platform, it took about 2 minutes to run the example data file provided in the tool folder.
6. Once successfully finished, the most bottom button “open result folder” shall be enabled.
7. Find results by press the “open result folder”. All quantitation results were provided in a .csv (comma separated) format file. All annotated spectra were provided in the subfolder.

# **Trouble shooting**

- 1. If the tool cannot be launched, firstly check whether all requirements have been successfully installed (python 2.7 version, module listed in section3). Test it by start python and import distinct modules and check the exact versions
  2. If you had any question on the usage of gQuant, kindly contact us via emails below: [jiangmingh@gmail.com](mailto:jiangmingh@gmail.com), [12110220006@fudan.edu.cn](mailto:12110220006@fudan.edu.cn) or [wqcao@fudan.edu.cn](mailto:wqcao@fudan.edu.cn). Better attach with some of screenshots of error message, if convenient.
